# A stem cell gene expression profile of human squamous cell carcinomas

**DOI:** 10.1016/j.canlet.2008.06.014

**Published:** 2008-12-08

**Authors:** Kim B. Jensen, Judith Jones, Fiona M. Watt

**Affiliations:** a Wellcome Trust Centre for Stem Cell Research, University of Cambridge, Tennis Court Road, Cambridge, CB2 1QR, United Kingdom; b Department of Oral & Maxillofacial Surgery, Guy’s Hospital, London SE1 9RT, United Kingdom; c CR UK Cambridge Research Institute, Li Ka Shing Centre, Robinson Way, Cambridge CB2 0RE, United Kingdom

**Keywords:** Squamous cell carcinoma, Stem cells, Epidermis, Keratinocyte, Cancer stem cell

## Abstract

To investigate the relationship between stem cells in normal epithelium and in squamous cell carcinomas (SCCs), we examined expression of a panel of human epidermal stem cell markers in SCCs and SCC cell lines. Markers that are co-expressed in normal stem cells were not co-expressed in SCC. Downregulation of two markers, Lrig1 and MAP4, and upregulation of a third, MCSP, correlated with poor differentiation status and increased proliferation in primary tumours. We conclude that SCCs do not reflect a simple expansion of stem cells; rather, tumour cells hijack the homeostatic controls that operate in normal stem cells, eliminating those that maintain stem cell quiescence.

## 1 Introduction

Stratified squamous epithelia act as a protective interface between the body and the environment. They have a simple organisation: proliferation takes place in the basal layer of cells attached to an underlying basement membrane, and cells undergo terminal differentiation as they move towards the tissue surface [1]. The outermost cell layers are shed throughout adult life and are replaced through proliferation of a subpopulation of cells in the basal layer known as stem cells [1]. Examples of stratified squamous epithelia are the epidermis, the lining of the oral cavity, the cervix and oesophagus.

One of the characteristic tumours of stratified squamous epithelia is squamous cell carcinoma (SCC). These tumours retain hallmarks of the normal epithelial terminal differentiation programme; however, proliferation is increased, the proportion of differentiated cells is decreased, and the spatial organisation of the cell layers is disrupted [1]. There is evidence that SCCs are maintained by a subpopulation of proliferative cells termed cancer stem cells, which are responsible for tumour maintenance and for regrowth following conventional treatment [2,3].

At present little is known about the relationship between stem cells in normal stratified squamous epithelia and stem cells in SCCs. However, it is generally believed that oncogenic changes in stem cells are responsible for SCC development because stem cells are the only long-lived cells within stratified epithelia [1]. We recently generated single cell cDNA libraries from normal human epidermal cells and used them to identify a panel of 14 stem cell markers [4]. In the present report, we have investigated their expression in SCC, with a view to discovering the relationship between stem cells in normal stratified squamous epithelia and SCC.

## 2 Materials and methods

### 2.1 Cell culture conditions and stem cell fractionation

Primary human keratinocytes from neonatal foreskin (strains kj, kt and kw), adult oral mucosa (strain OK) and the SCC lines SCC9, SCC12B.2, SCC13, SCC15, SCC25, SCC27 [5,6] and H376 [7] were cultured in the presence of Mitomycin-C treated J2-3T3 feeder cells as described previously [8]. Stocks of A431 cells were cultured in DMEM supplemented with 10% bovine serum, penicillin and streptomycin. In clonal growth assays all cells, including A431 cells, were cultured with a feeder layer in the standard keratinocyte medium. For RNA and protein isolation the medium of all cultures was changed the day before analysis to fresh standard keratinocyte medium.

Cell fractionation by rapid adhesion to 40 μg/ml type I collagen was performed as described previously [4,9]. For cell sorting, cells were labelled with Alexa 647 anti-human β1-integrin antibody (Serotec), washed and subjected to flow cytometry using a Dako MoFlo high-speed cell sorter. Fractions of 100 cells were collected from the 10% of cells expressing highest (β1^high^) and lowest (β1^low^) levels of β1-integrin in addition to all β1-integrin positive cells (All sort) and subjected to clonal analysis as described previously [9].

### 2.2 Real-time PCR analysis

RNA was isolated from subconfluent cultures of cells using Tri-reagent (Invitrogen) and cDNA was generated as described previously [4]. Quantitative-PCR was performed according to the manufacturer’s instructions. The list of genes examined is described in Supplementary material.

### 2.3 Immunostaining and antibodies

Human oral SCCs, normal oral mucosa and normal skin were obtained with appropriate ethical approval. Frozen tissue sections were fixed in 0.5% paraformaldehyde for 5 min and permeabilised for 5 min with 0.4% Triton X-100. After blocking with 10% FCS in PBS, sections were incubated for 1 h with antibodies diluted in 10% FCS in PBS. Cells and sections were washed with 10% FCS in PBS between incubations with primary and secondary antibodies. Antibodies were used against the following antigens: MCSP, MAP4 (9.2.27 and clone 18, BD Life Sciences), Lrig1 (gift from Dr. Satoshi Itami) and Ki67 (NCL-L-Ki67-MM1, Novocastra). Secondary antibodies conjugated with Alexa Fluor 488 or 594 were obtained from Invitrogen.

Sections were mounted with Moviol and examined using a Zeiss 510 confocal microscope. For quantitative measurements sections were examined with a 20× objective using the Ariol^®^ system manufactured by Applied Imaging. This generated a numerical value that reflected both staining intensity and the proportion of tissue with detectable staining.

## 3 Results

### 3.1 Expression of stem cell markers in human squamous cell carcinoma lines

We used real-time quantitative PCR (Q-PCR) to examine gene expression levels of the 14 recently described human epidermal stem cell markers in a panel of human SCC cell lines. Five lines were derived from tumours of the oral cavity (SCC9, SCC15, SCC25, SCC27 and H376), two from facial epidermis (SCC12B.2 and SCC13) and one from the vulva (A431). RNA was also isolated from primary keratinocytes derived from normal human epidermis or oral mucosa.

**Fig. 1:**
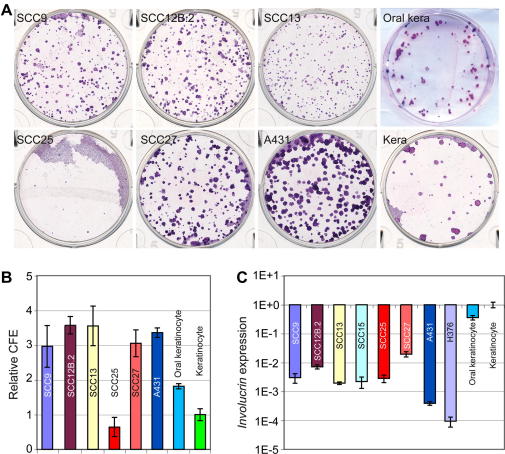
Clonal growth and differentiation of human SCC lines. (A) Representative dishes of cells grown at clonal density and stained with Rhodanile B are shown. (B) Quantitation of clonal growth was based on triplicate dishes in three independent experiments ± standard deviation. (C) Analysis of *Involucrin* expression. Expression levels of *Involucrin* were determined by Q-PCR and normalised to expression of *18S rRNA*. Keratinocyte: epidermal keratinocytes.

The SCC lines represented a range of ability to proliferate and differentiate in culture (Fig. 1) [5–7,10]. All lines except for SCC25 showed a 2- to 3-fold increase in colony forming efficiency and expressed lower levels of the differentiation marker, *involucrin*, compared to epidermal and oral keratinocytes (Fig. 1). Thus, by the criteria of increased clonal growth ability and reduced involucrin expression [9] the SCC lines appear to contain a higher proportion of stem cells than primary keratinocytes.

**Fig. 2:**
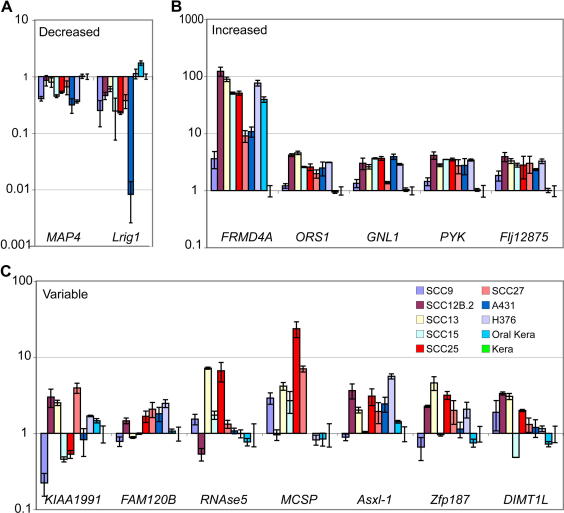
Analysis of levels of stem cell marker genes in by quantitative PCR. (A–C) Q-PCR of normal epidermal keratinocytes (Kera), oral keratinocytes (Oral kera) and SCC lines. Expression levels of stem cell markers were normalised to expression of *18S rRNA*. Genes were assessed for statistically significant differences against the panel of cell lines (unfractionated) and genes categorised as either down (A) or up (B) regulated if the *P*-value was less than 0.01. (C) Genes with variable expression levels. Error bars represent the standard error of the mean for at least triplicate samples.

We used Q-PCR to examine the expression of the 14 recently described human epidermal stem cell markers [4]. Since most of the SCC lines were derived from the oral cavity, we also compared marker expression in normal oral and epidermal keratinocytes. Marker expression in keratinocytes from the two sites was similar, except for *FRMD4A*, an ERM domain-containing protein involved in cytoskeleton rearrangement, which was expressed at higher levels by oral keratinocytes (Fig. 2).

The markers could be assigned to three categories, according to whether expression was decreased, increased, or variable in SCC lines compared to normal epidermal keratinocytes (Fig. 2). The two markers that were consistently downregulated in the SCC lines were microtubule-associated protein 4 (MAP4) and Leucine-rich repeats and immunoglobulin-like domains 1 (Lrig1) (Fig. 2A). MAP4 is reported to regulate cell cycle progression and to be ubiquitously expressed in proliferating cells [11], whereas Lrig1 is a negative regulator of the EGF receptor and mediates epidermal stem cell quiescence [4,12,13].

The markers that were consistently upregulated in SCC lines were: *FRMD4A*; oxidative stress response gene 1 (*OSR1* or *C20orf111*); guanidine nucleotide binding protein 1-like protein (*GNL1* or *HSR1*), which is another stress response gene; a serine kinase, phosphorylase b kinase (*PYK*), and *FLJ12875*, a regulator of the I-kappaB kinase/NF-kappaB cascade. The upregulation was most striking in the case of *FRMD4A* (Fig. 2B); however, since expression was high in normal oral keratinocytes we conclude that elevated *FRMD4A* expression may reflect the site of origin rather than the malignant status of the cells.

The final category of markers showed variable expression among the SCC lines (Fig. 2C). These were the putative ring finger protein *KIAA1991*; *FAM120B*, a protein containing domains associated with DNA repair; *Rnase5*, which is involved in mRNA degradation; the cell surface proteoglycan *MCSP*; Additional Sex Combs-like 1 (*Asxl-1*), which modulates polycomb and trithorax activity in *Drosophila*; a putative transcription factor, zinc finger protein 187 (*Zfp187)*; and dimethyladenosine transferase 1-like (*DIMT1L*), which regulates ribosome activity. It is worth noting that although MCSP is in the ‘variable’ category, it was upregulated in five out of seven primary SCC lines from oral and facial epithelia.

### 3.2 Isolation of clonogenic SCC cells

**Fig. 3:**
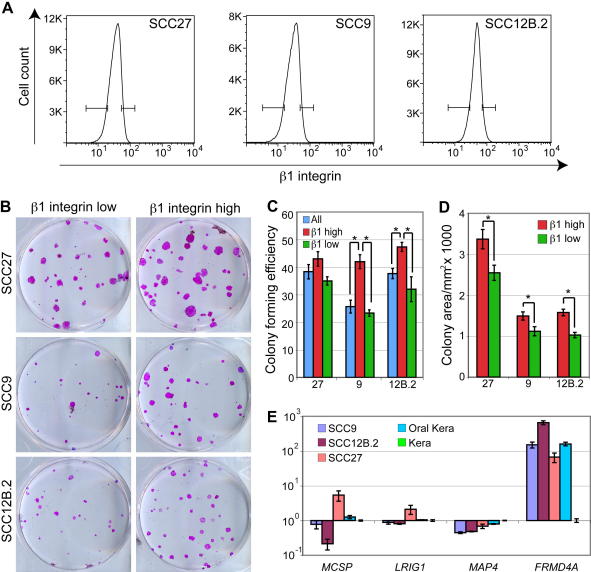
Selection of clonogenic SCC cells. (A) Basal cells, gated on the basis of low forward and side scatter [9] were labelled with anti-β1-integrin antibody. The 10% of cells with highest (β1-integrin high) and lowest (β1-integrin low) integrin levels were sorted and plated out at clonal density. (B) Representative dishes of cells from the clonogenicity assay, stained with Rhodanile B. (C) Colony forming efficiency was determined as % plated cells that formed colonies. ±SEM of triplicate samples. Asterisks indicate significant differences (*p* < 0.05, two-way unpaired *T*-test). (D) Relative colony size was measured using Image J, and the mean was determined for all colonies ± SEM. Asterisks indicate significant differences (*p* < 0.05, two tailed Mann–Whitney test of the median). (E) Q-PCR for *MCSP*, *MAP4*, *Lrig1* and *Frmd4a* in populations of normal oral and epidermal keratinocytes and a subset of SCC lines enriched for stem cells by rapid adhesion to collagen.

Human epidermal stem cells can be enriched by selecting the basal cells that express the highest levels of β1-integrins and adhere most rapidly to collagen [9]. The SCC lines examined express similar β1-integrin levels to normal oral and epidermal keratinocytes [14,15]. We therefore investigated whether we could enrich for clonogenic SCC cells on the basis of adhesiveness. In the case of SCC9 and SCC12B.2, the 10% of cells with the highest β1-integrin levels (β1^high^) had a significantly higher colony forming efficiency than all sorted cells and the 10% of cells with the lowest levels (β1^low^) cells (Fig. 3B and C). This was not observed for SCC27; however the colonies formed by β1^high^ SCC27 cells were larger than colonies formed by β1^low^ cells (Fig. 3D). Thus, as in normal epidermal keratinocytes, it is possible to enrich for clonogenic SCCs on the basis of high β1-integrin expression.

To investigate whether the observed expression patterns in the SCC derived lines reflected variation within the undifferentiated, clonogenic cells, we performed Q-PCR on cDNA generated from cells fractionated on the basis of rapid adhesion to collagen, which reflects high β1-integrin levels (Fig. 3; [9]). In SCC9, SCC12B.2 and SCC27 cells and oral keratinocytes the relative levels of marker expression when compared to epidermal keratinocytes were largely the same as in unfractionated cells (Fig. 3E). FRMD4A was upregulated in oral keratinocytes and SCC cells compared to epidermal keratinocytes. MAP4 expression was reduced in all the SCC lines and MCSP expression was variable, expression being lowest in SCC12B.2 and highest in SCC27, reflecting the changes observed in unfractionated cells. However, whereas reduced Lrig1 expression was a consistent characteristic of unfractionated cells (Fig. 2A), the levels of Lrig1 were remarkably similar in the enriched populations (Fig. 3D). Thus, clonogenic cells in SCC lines and primary keratinocytes express similar levels of Lrig1. The decrease in Lrig1 expression that characterises SCC lines may therefore be associated with expansion of the transit amplifying compartment.

### 3.3 Studies in primary tumours

The data from the SCC lines make two predictions about marker expression in primary tumours: that markers that are co-expressed in the basal layer of normal epidermis are not co-expressed in SCCs; and that downregulation of MAP4 and Lrig1 is a characteristic feature of SCCs. Support for the second prediction came from analysis of a publicly available gene data set deposited at Oncomine [16,17]. We found that MAP4 and Lrig1 were significantly downregulated in oral SCCs, while CD44, which has recently been reported to be a marker of tumour initiating cells in head and neck SCCs [3], and MCSP were highly upregulated (Table 1).

To examine the sites of marker expression, we stained sections of 18 human oral SCCs, one adenoid cystic carcinoma, one lymph node metastasis of an oral SCC and two specimens of normal skin, as well as four samples of normal oral mucosa. We evaluated proliferative status by Ki67 labelling and graded the differentiation status of the tumours according to standard criteria [18] (Table 2).

Expression levels in normal and tumour tissue were scored by eye as negative (−/+), weak (+), moderate (++) or strong (+++), based on the intensity and extent of staining (Table 2). We confirmed our scoring for Lrig1 expression on one sample of normal skin and five tumours, picked at random using an Applied Imaging Ariol^®^ system. The results were as follows (visual score in brackets): normal epidermis, 78 ± 3 (+++); tumour W3, 49 ± 6 (++); tumour P3, 21 ± 3 (+/−); tumour M4, 32 ± 12 (+); tumour M7, 33 ± 10 (+/−); tumour MET1, 17 ± 7 (−). There was thus good agreement between the visual and automated scores.

**Fig. 4:**
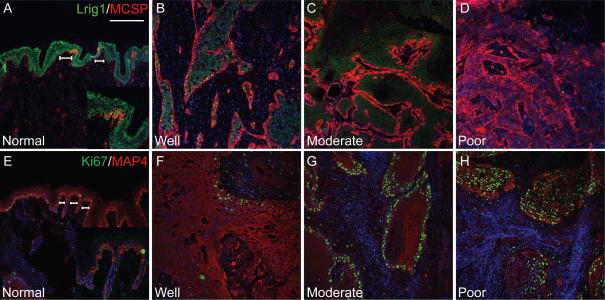
Expression of stem cell markers in human epidermis and squamous cell carcinomas. Expression of (A–D) MCSP (red) and Lrig1 (green), (E–H) MAP4 (red) and Ki67 (green) in epidermis (normal) and in squamous cell carcinomas of differing differentiation status (well, moderate, poor). Location of putative stem clusters is marked with horizontal lines. Panels A, E contain higher magnification inserts. Scale bars: 200 μm.

As reported previously [4], in normal epidermis Lrig1 and MCSP were expressed most abundantly in clusters of putative stem cells in the basal layer (brackets in Fig. 4A). MAP4 was expressed throughout the epidermal basal layer, expression being highest in the stem cell clusters (brackets in Fig. 4E). Thus, in normal epidermis Lrig1, MCSP and MAP4 were co-expressed, expression being highest in the putative SC clusters that are known to be less frequently dividing than other cells in the basal layer [19,20].

As predicted, expression of Lrig1 and MAP4 was markedly decreased in tumours (Fig. 4B–D, F–H and Table 2). The number of cells expressing Lrig1 was reduced, as was the labelling intensity (Fig. 4B–D). In tumours, there was a general reduction in MAP4 staining intensity, irrespective of differentiation status (Fig. 4F–H). Moreover, low levels of MAP4 expression extended throughout the tumour mass, whereas in normal epidermis expression was confined to the basal layer.

There was a tight correlation between expression of Lrig1 and the degree of tumour differentiation, as reported previously [21]. Lrig1 was detected in well and moderately differentiated tumours but was markedly reduced in poorly differentiated tumours (Fig. 4B–D). In 4/5 poorly differentiated tumours Lrig1 was either undetectable (−) or expressed in only occasional cells (−/+ or +). In one tumour most areas had only a few positive cells (+), although there was one region with more extensive staining (++). Ki67 positive cells did not express Lrig1, consistent with its role as a negative regulator of proliferation (data not shown). In well and moderately differentiated tumours Lrig1 was detected in the suprabasal layers, correlating with areas that were Ki67 negative and consistent with Lrig1 expression in normal oral mucosa (data not shown, Table 2).

While Lrig1 and MAP4 expression was decreased in tumours, MCSP expression was upregulated (Table 2; Fig. 4B–D). In well and moderately differentiated tumours MCSP expression was largely confined to the epithelial layer adjacent to the tumour stroma (Fig. 4B and C). In poorly differentiated tumours MCSP was expressed throughout the tumour (Fig. 4D). Whereas Lrig1 and MCSP are co-expressed in the basal layer of normal epidermis ([4]; Fig. 4A), MCSP positive cells in tumours lacked Lrig1 expression (Fig. 4B–D).

## 4 Discussion

The panel of markers that were co-expressed in keratinocytes from normal human epidermis [4] were, with the exception of FRMD4A, expressed at similar levels in keratinocyte from normal oral mucosa. Furthermore, we could enrich for clonogenic, putative stem cells on the basis of high β1-integrin expression and rapid adhesion to collagen in normal epidermal and oral keratinocytes, and in some of the SCC lines examined. While we did not examine inter-clonal differences within the SCC lines [2], the overall increase in colony forming efficiency and decrease in *involucrin* expression would indicate an expansion of the stem cell compartment. Nevertheless, these features did not correlate with increased expression of the entire panel of stem cell markers. Instead, while some markers were upregulated, two were downregulated and the remainder showed variable changes amongst the different SCC lines. In addition, basal cells in SCCs did not maintain the full complement of markers normally observed in the basal layer of oral and epidermal epithelium. Thus, there is no evidence for expansion of the normal stem cell compartment in SCCs.

Downregulation of MAP4 and Lrig1 and upregulation of MCSP were consistent features of SCC lines and primary tumours. Lrig1 is known to negatively regulate EGFR signalling, thereby maintaining epidermal stem cells in a quiescent, nondividing state [4,21]. It is likely that loss of Lrig1 in SCCs, which is associated with a poor prognosis [21,22], will confer a significant growth advantage in the basal layer, and lead to an expansion of committed progenitors via upregulation of EGFR signalling. Just as loss of Lrig1 will impact on a variety of signalling pathways, so will upregulation of MCSP. MCSP activates the small GTPases CDC42 and Rac1 via an integrin-mediated pathway [23,24]. This is intriguing as we previously reported increased expression of the small GTPase Rac1 in SCCs as well as the tumour promoting role of altered epidermal integrin expression [25,26]. Less is known about the likely consequences of loss of MAP4 in SCCs. MAP4 undergoes extensive posttranslational modifications and interacts with a number of different proteins that regulate cell cycle progression and cytokinesis [11]. We speculate that reduced levels of MAP4 in SCCs facilitate more rapid cell cycle progression; however, this remains to be tested.

We have demonstrated the existence of cellular heterogeneity in SCC lines, based on the expression of β1 integrins and ability to adhere to extracellular matrix components. In three out of four genes analysed, differences in expression observed in the clonogenic cell-enriched fraction reflected differences in the total cell population. The notable exception was Lrig1. While Lrig1 was consistently downregulated in unfractionated SCC lines and primary tumours, the levels of Lrig1 in the stem cell-enriched fractions were remarkably similar to normal oral and epidermal stem cells. This suggests that there is a subpopulation of SCC cells that are subject to negative regulation of EGFR signalling. Their existence is important clinically, because these cells will be less likely to respond to drugs that target cells with elevated EGFR signalling and may be capable of reinitiating tumour growth following therapy. In addition, it will be important to determine whether tumour initiation is a general feature of basal cells with high β1-integrin levels, or whether a combination of markers such as CD44, MCSP and Lrig1 will allow further enrichment.

In conclusion, our data favour a model whereby during tumour development the pathways that control epithelial homeostasis are lost, particularly in the basal cell layer closest to the tumour stroma. Those markers of normal stem cells that exert a positive effect on proliferation or inhibit differentiation are upregulated, while those that normally retain the cells in a nondividing state show reduced expression. As we find out more about how different signalling pathways intersect to maintain homeostasis we will have more opportunities for restoring homeostasis in tumours.

## Conflict of interest

The authors declare no conflict of interest.

## Figures and Tables

**Table 1:** A panel of selected genes known to be either involved in tumour progression and/or SC maintenance in normal tissue

Gene name	Entries	Signal	*P*-value
CD44	3	Up	6.70 E−14
MCSP	1	Up	2.50 E−04
MAP4	1	Down	5.10 E−06
LRIG1	2	Down	2.60 E−05

The data are derived from expression profiling of 31 oral squamous cell carcinomas and 26 samples of normal oral epithelium [17]. The gene name is listed in column 1, the number of different probes detecting the gene is indicated in column 2, whether the gene is up or downregulated is listed in column 3 and the *P*-value for the finding is listed in column 4.

**Table 2:** Expression pattern of selected proteins in human primary SCCs as determined by indirect immunofluorescence

Tumour	Lrig1	MCSP	MAP4	Differentiation	Tissue
Foreskin	+++	+	+++		
Oral mucosa	++	+	+++		
P1	+	++	+/++	Poor	Lateral border of tongue
P2	+	++	+	Poor	Tongue
P3	−/+	+++	+	Poor	Maxillary antrum
P4	+/++	+++	++/ +++	Poor	Buccal mucosa
P5	−	+	+/++	Poor	Right tonsil
MP1	++	++	++	Mod/poor	Dorsum tongue
M1	+	++	+	Moderate	Lateral border of tongue
M2	+	++	+	Moderate	Retromolar
M3	++	++	++	Moderate	Lateral border of tongue
M4	+	+++	+/++	Moderate	Lateral border of tongue
M5	+	++	+/−	Moderate	Alveolar ridge maxilla
M6	+	++	+	Moderate	Buccal mucosa
M7	−/+	+++	+	Moderate	Buccal mucosa
M8	++	+++	+	Moderate	Lateral tongue
W1	++	++	+	Well	Lateral border of tongue
W2	++	+++	+/++	Well	Buccal mucosa
W3	++	++	+	Well	Ventral tongue
W4	++	++	+	Well	Floor of mouth
ACC1	—	+++	−/+	Adenoid cystic carcinoma	Sublingual gland
MET1	—	++	—	Poor	Submandibular lymph node
*Summary*					
Poor	Down	Up	Down		
Moderate	Down	Up	Down		
Well	NGC	Up	Down		

Staining intensity was scored by eye as negative (−/+), weak (+), moderate (++) or strong (+++), based on the intensity and extent of staining. NGC: no general change.
