# Supplementary material for: A stem cell gene expression profile of human squamous cell carcinomas
Source: Cancer Lett. 2008 Dec 8;272(1):23–31. doi: 10.1016/j.canlet.2008.06.014 (PMC2580815; doi:10.1016/j.canlet.2008.06.014)
Supplement: Supplementary material [file mmc1.doc]

**Supplementary material:**

A stem cell gene expression profile of human squamous cell carcinomas

Kim B. Jensen, Judith Jones and Fiona M. Watt

**Real time PCR analysis of stem cell genes**

Quantitative-PCR was performed according to manufacturers instructions for genes *GAPDH* (Hs99999905_m1, NM_002046.3), *18S ribosomal rRNA* (Hs99999901_s1, X03205.1), *Involucrin* (Hs00846307_s1, NM_005547.2)*, Melanoma Chondroitin Sulphate Proteoglycan (MCSP)* (Hs00426981_m1, NM_001897.3), *LRIG1* (Hs00394267_m1, NM_015541.2), *KIAA1991* (Hs01397828_m1, AB082522.1), *FAM120B* (Hs00921806_m1, NM_032448.1), *Asxl-1* (Hs_00392415_m1, NM_015338.3), *FERM4D* (Hs00216036_m1, NM_018027.2), *Corf20-111* (Hs00212852_m1, NM_016470.6), *GNL1* (Hs01073513_g1, NM_005275.2), *PYK* (Hs00175999_m1, NM_000292.1), *RNase5* (Hs_00268002_s1, NM_194430.1), *FLJ12875* (Hs00226069_m1, NM_024544.1), *MAP4* (Hs00159048_m1, NM_030884.2), *DIMT1L* (Hs00205515_m1, NM_014473.2) and *ZFP187* (Hs00939647_m1, NM_152736.3) (all purchased from Applied Biosystems). Real-time PCR reactions and analysis were performed with an ABI Prism 7700 and an ABI Prism HT7900 Sequence Detection System (Applied Biosystems). The relative amount of each mRNA was normalized to the levels of *18S rRNA*.
